# Errors within the total laboratory testing process, from test selection to medical decision-making – A review of causes, consequences, surveillance and solutions

**DOI:** 10.11613/BM.2020.020502

**Published:** 2020-06-15

**Authors:** Cornelia Mrazek, Giuseppe Lippi, Martin H Keppel, Thomas K Felder, Hannes Oberkofler, Elisabeth Haschke-Becher, Janne Cadamuro

**Affiliations:** 1 Department of Laboratory Medicine, Paracelsus Medical University, Salzburg, Austria; 2 Section of Clinical Chemistry, University of Verona, Verona, Italy

**Keywords:** total testing process, extra-analytical phase, quality indicators, laboratory medicine, patient safety

## Abstract

Laboratory analyses are crucial for diagnosis, follow-up and treatment decisions. Since mistakes in every step of the total testing process may potentially affect patient safety, a broad knowledge and systematic assessment of laboratory errors is essential for future improvement. In this review, we aim to discuss the types and frequencies of potential errors in the total testing process, quality management options, as well as tentative solutions for improvement. Unlike most currently available reviews on this topic, we also include errors in test-selection, reporting and interpretation/action of test results. We believe that laboratory specialists will need to refocus on many process steps belonging to the extra-analytical phases, intensifying collaborations with clinicians and supporting test selection and interpretation. This would hopefully lead to substantial improvements in these activities, but may also bring more value to the role of laboratory specialists within the health care setting.

## Introduction

The modern health care is inevitably dependent on laboratory results for diagnosis, prognosis and/or treatment decisions (*1*). Therefore, accurate performance of all the steps included within the traditional brain-to-brain loop, *i.e.*, test ordering/test-selection, sample collection, identification, transport, sample preparation, analysis, test reporting, interpretation and action is important (*2*).

Unfortunately, each of these steps is vulnerable to errors, which can then potentially generate erroneous results and finally jeopardize patient safety. To mention only a few examples, the specimen may be drawn from the wrong patient; erroneous low calcium and alkaline phosphatase may be misinterpreted when potassium-ethylenediaminetetraacetic acid (K-EDTA) contamination is not identified; pseudohyperkalaemia due to extreme leucocytosis may lead to unnecessary and even potentially dangerous treatment (*3*-*5*).

There is now incontrovertible evidence that the vast majority of laboratory errors occur in the preanalytical phase (61.9 - 68.2%), which are then followed by mistakes in the postanalytical (18.5 - 23.1%) and analytical (13.3 - 15%) parts of the total testing process (TTP) (*6*, *7*). Using the same study design in 1996 and 2006, Carraro and Plebani attributed the decline of the error rate of samples contaminated by infusion fluids from 20.6% to 1.9% to corrective actions. Together with the statement that 73% of errors in the TTP seem to be preventable, this reinforces the need of vigilance and monitoring of laboratory vulnerability (*7*).

**Figure 1:**
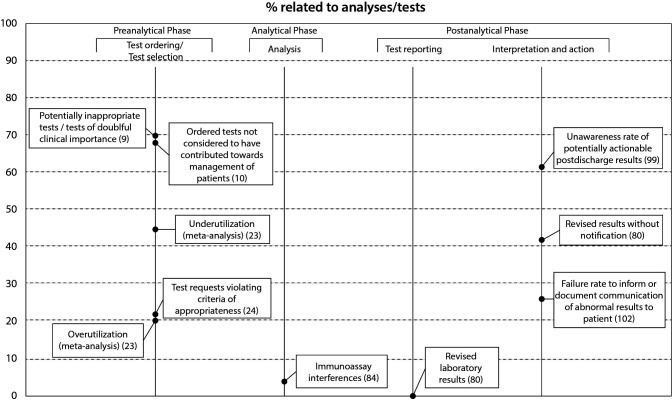
Published data on error rates (reference numbers are indicated in brackets) related to analyses/tests.

**Figure 2:**
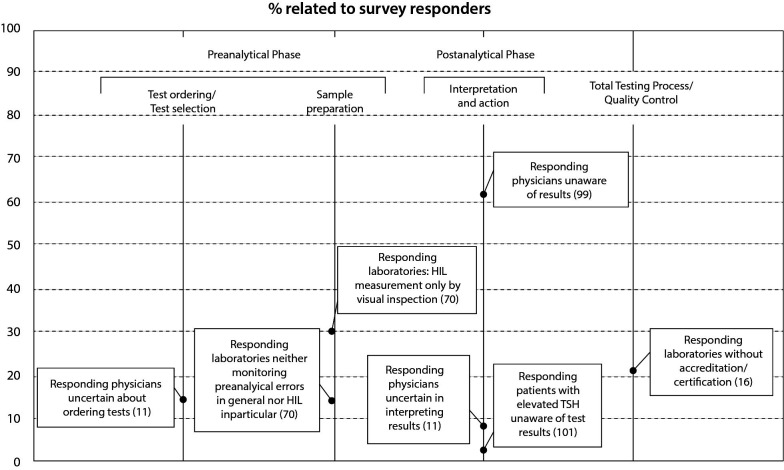
Published data on error rates (reference numbers are indicated in brackets) related to survey responders. HIL - haemolysis, icterus, lipemia.

**Figure 3:**
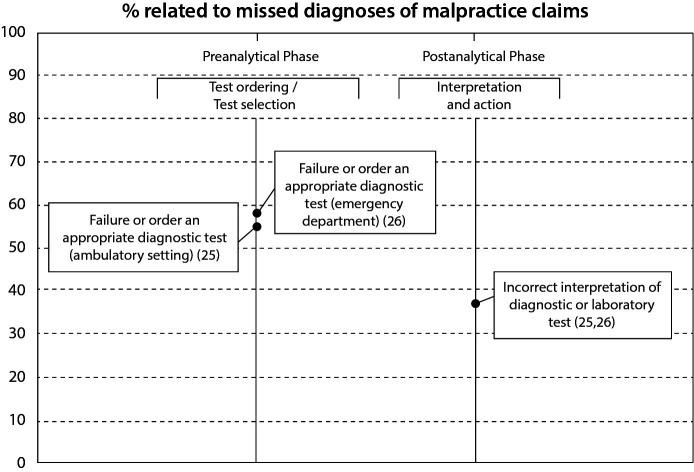
Published data on error rates (reference numbers are indicated in brackets) related to missed diagnoses of malpractice claims.

**Figure 4:**
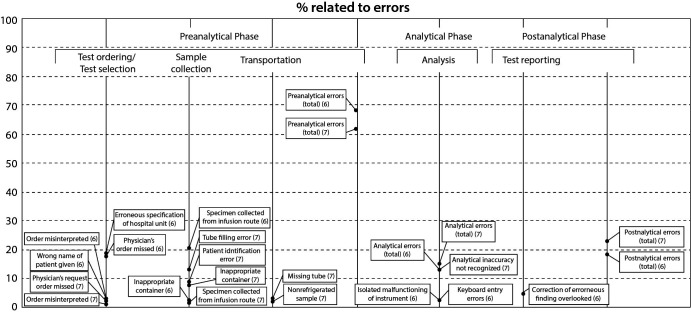
Published data on error rates (reference numbers are indicated in brackets) related to errors.

**Figure 5:**
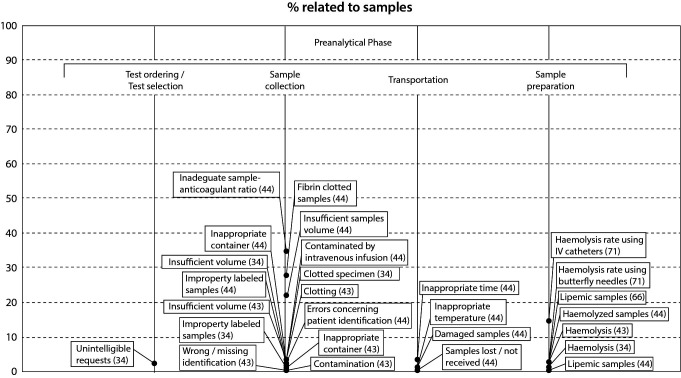
Published data on error rates (reference numbers are indicated in brackets) related to samples. IV - intravenous.

**Figure 6:**
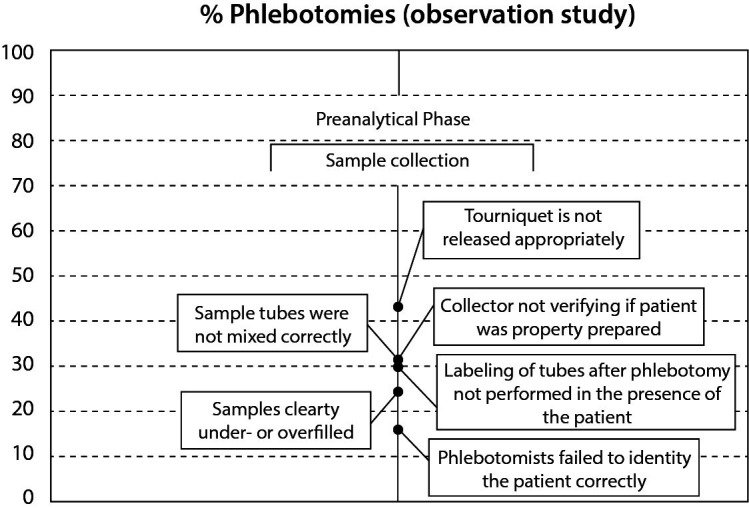
Error rates related to phlebotomies in an observational study (*42*).

As error rates are traditionally reported from blood collection to result reporting, less emphasis has been given to appropriateness in test selection, result interpretation and medical action, phases, some authors refer to as “pre-pre”- and “post-post”- analytical phase (*8*). For an easier understanding, we will refrain from using these terms, since respective processes may be subsumed under the pre- or postanalytical phases. However, laboratory specialists must not neglect these steps of the TTP, whereby many studies show high frequencies of inappropriate test selection and uncertainty in result interpretation (*9*-*11*). Moreover, inappropriate test selection seems to be especially more frequent than all other errors that have been identified so far (Figure 1). In this review, we hence want to describe the types and frequencies of errors, which may occur during the TTP (*i.e.*, the brain-to-brain loop), including test selection and interpretation/medical action. Due to different study designs, frequencies of errors are related to heterogeneously acquired data and are therefore not entirely comparable. Nevertheless, to get an overview of the numbers mentioned in the review, we plotted them in figures, separated in percentages related to analyses/tests, survey responders, missed diagnoses of malpractice claims, errors, samples, and phlebotomies of an observational study (Figure 1-6).

Moreover, we aim to provide some suggestions on how these errors can be measured and we will mention some tentative strategies for improvement. In order to provide a quick overview, we additionally summarized these issues in a supplemental table (Appendix 1), categorized by the TTP phase, the TTP step and the sources of error, including respective quality indicators (QI) measurement options as well as possible solutions for improvement.

## Quality control

Following the Plan-Do-Check-Act (PDCA) cycle is a widely used tool to improve certain processes (*12*). Quality control as the “check”-part thereof is essential to detect error-prone stages of the TTP, which need further improvement activities. The low error rates in the intra-laboratory parts of the TTP can be attributable to the fact that these are under strict control of highly trained laboratory personnel. In addition, the vast majority of laboratories have now implemented a quality management system according to the requirements of ISO 15189, ISO 9001, or other national standards (*13*, *14*). An interesting relationship has been published by Buchta *et al.*, who showed that laboratories using an immunohaematology external quality assessment (EQA) scheme with ISO 9001 certification or ISO 15189 accreditation have lower error rates than others (*15*). However, further studies are needed to evaluate the improvement of error rates by the use of quality management systems. In a recent survey 21% of participating European laboratories admitted that they were not accredited or certified (Figure 2) (*16*).

In analogy with the analytical phase, more attention should be paid to quality assessment in the pre- and postanalytical phase (*17*). One possibility is participating in the model of QIs program, which has been established by the International Federation of Clinical Chemistry and Laboratory Medicine (IFCC) Working Group “Laboratory Errors and Patient Safety” (WG-LEPS) and the European Federation of Clinical Chemistry and Laboratory Medicine (EFLM) Task and Finish Group “Performance specifications for the extra-analytical phases” (TFG-PSEP). Laboratories can log in for free through the web-portal (www.ifcc-mqi.com), enter local process-specific QIs and benchmark them to other national and international laboratories (*18*). The proposed QIs, summarized in Appendix 1, span throughout the TTP. Notably, additional programs have been established at the national level, like the German/Austrian Preanalytical Benchmark Database for comparison of haemolysis data (*19*). The Six Sigma approach would be another way to document and compare errors (*20*). In addition, the defects *per* million opportunities (DPMO) should be stated. An improvement of the sigma value from three to four would correspond to a change in DPMO from 66,800 to 6200 and 2700 to 63 for long-term and short-term Sigma metric, respectively (*21*).

Despite these efforts, a recent survey among European laboratories revealed that although the majority of laboratories already document/monitor preanalytical errors, about a third of them fail to evaluate their data and, even when a statistical analysis is made, approximately 25% of them remain inactive against unsatisfactory results (*16*).

## Test ordering/test selection

A recent survey carried out among 1347 European laboratories, categorized responses to the open-ended question “Which preanalytical topics concern you most?” into three topics: analyte stability, analytical interference (haemolysis, icterus, lipemia (HIL)), and compliance to venous specimen collection guidelines (*16*). This would hence lead to conclude that laboratories are still focusing on the TTP from “Sample collection” onwards, thus overlooking the first and essential step “Test ordering/test selection”, where laboratory specialists could initiate collaborations with clinicians to overcome inappropriate test requesting habits.

According to the “five rights rule” paradigm, an effective strategy for preventing errors encompasses that the right test must be ordered in the right patient at the right time (*22*). Evidence that this practice is not thoughtfully followed comes from a survey among 1768 primary care physicians, which revealed uncertainty on test ordering in as many as 15% of respondents (Figure 2) (*11*).

Inappropriate use of laboratory tests may present as over- or underutilization. Reasons for overutilization – *i.e.*, ordering tests which are not appropriate - may include the use of routine laboratory ordering panels, non-adherence to re-testing intervals or biological implausibility (*9*, *23*, *24*). An interesting meta-analysis has recently shown that the mean rates of overutilization can be as high as 20.6% (Figure 1) (*23*). Nevertheless, up to 70% of requests may have questionable clinical significance in single studies (*9*, *10*).

Underutilization, *i.e.*, failure to order the correct diagnostic test, is comprised within the leading causes of missed or delayed diagnoses, and should hence be considered a major threat of patient safety (*23*). In closed malpractice claims, underutilization has been identified as a major contributor of missed and delayed diagnoses in up to 55% ambulatory cases, and in up to 58% emergency department cases, respectively (Figure 3) (*25*, *26*). With the limitation of the small number of studies addressing this issue, a meta-analysis published by Zhi *et al.* concluded that the mean rates of inappropriate underutilization of laboratory tests can approximate 45% (*23*).

Specialists in laboratory medicine should acknowledge inappropriate test requests by measuring the corresponding QIs (*18*). Appropriate solutions may be developed in collaboration with clinicians for reducing the number of inappropriate test requests, by applying one or more efficient strategies, which may include education through feedback, use of interpretive comments, automated flags when tests have no clinical value in the specific setting or are repeated too early, gate-keeping strategies for tests with a high negative predictive value, diagnostic pathways (*i.e.*, “algorithms”) for specific indications/symptoms, implementation of reflex criteria for defined pathological test results, reflective testing or establishment of diagnostic management teams (*8*, *10*, *24*, *27*-*32*).

Additional errors within the ordering process may occur during the test requesting procedure itself. The data entry into the hospital information system (HIS) may be incorrect, or the wrong patient may be selected. Test requests can also be misinterpreted, unintelligible or get lost (Figures 4-5) (*6*, *7*, *33*, *34*). The use of computerized physician order entry (CPOE) systems coupled with barcodes for patients and blood tubes are advisable for reducing the intrinsic risks associated with paper-based test requests (*33*, *35*).

## Sample collection

In this section of the review we will focus on errors potentially occurring during venous blood sampling. A specific discussion on collection of capillary, cerebrospinal fluid, urine and blood culture samples ought to be omitted for space constrains, though information can be garnered elsewhere (*36*-*39*).

In 2018 the EFLM Working Group for Preanalytical Phase (WG-PRE) and the Latin American Working Group for the Preanalytical Phase (WG-PRE-LATAM) of the Latin America Confederation of Clinical Biochemistry (COLABIOCLI) have jointly issued a Consensus Guideline on venous blood collection, aiming to provide evidence-based guidance on every single step of the phlebotomy process (*40*).

### Patient identification and tube labelling

The accurate identification of the patient and the appropriate labelling of blood collection tubes are crucial steps for preventing diagnostic errors and inappropriate patient management.

Patient identification should be performed by asking open questions and/or comparing the patient’s identification (barcoded) bracelet using at least two identifiers (*33*, *41*). Collection tubes should be labelled directly before or after phlebotomy, but always in the presence of the patient. In an EFLM WG-PRE observational study, phlebotomists failed to identify the patient according to Clinical and Laboratory Standards Institute (CLSI) or local guidelines in up to 16% of cases. When sample tubes were labelled after phlebotomy, labelling was not carried out in the presence of the patient in nearly one-third of cases (Figure 6) (*42*). This evidence is then reflected by data on sample rejection for misidentification or receipt of unlabelled tubes, leading to rejection rates as high as 0.2% of all samples (Figure 5) (*34*, *43*, *44*). As a proportion of errors analysed, patient identification account for approximately 9% (Figure 4) (*7*).

Misidentification errors may be surveilled by QIs (*18*). Most possibilities aiming to reduce identification errors encompass some form of automation: barcoding system for identification and labelling, occasionally with automated systems for labelling of tubes or pre-labelled tubes (*33*, *35*, *45*).

### Patient preparation and time of blood collection

Collecting blood in a non-fasted state and even chewing of a sugar-free gum may influence laboratory parameters (*46*, *47*). Moreover, the concentration of specific analytes, *e.g.* catecholamines, may be influenced by the type of aliments ingested recently (*48*). Information on physical activity, as well as intake of drugs (time, dosage), are also important for the accurate interpretation of test results (*40*, *49*). Moreover, patients should rest for at least 15 minutes, either lying or sitting before blood collection (*40*, *49*, *50*). In special cases, *e.g.* catecholamines in plasma, these demands may be even more stringent (*48*).

In a recently published study, Simundic *et al.* reported that the phlebotomist failed to verify whether the patient was correctly prepared for blood collection in over 31% of samplings (*42*).

Blood samples should be collected in the morning, to prevent the impact of diurnal variation (*46*, *49*). The time of sample collection should always be documented, to verify whether laboratory analyses are performed within the time of stability of the respective parameter.

In selected cases (*e.g.* emergency or outpatient wards) adherence to these recommendations is not always possible in daily routine. Moreover, under defined circumstances, interpretation of certain parameters may even be possible (*e.g.* lipid results in a non-fasting state) (*51*). Nevertheless, information about the preparation of the patient should be documented to avoid misinterpretation of results (*40*).

### Sample contamination by intravenous infusion

Blood should never be drawn at the infusion site or proximal thereof. In case intravenous (IV) lines cannot be avoided for blood collection, the flushing of the line and the subsequent discard of a certain blood volume should be carried out correctly (*49*, *52*). The rate of samples rejected for contamination with fluids from intravenous infusions can be as high as 2.2% of overall samples (*44*).

### Tourniquet time

If venous stasis cannot be avoided, the tourniquet should be released within one minute while the blood is collected into the first tube to avoid alterations due to fluid shifts (*49*). In the EFLM WG-PRE observational study, it was found that the tourniquet is not released appropriately in 43% of observed blood collections (*42*). Furthermore, the phlebotomist has to advise the patient not to clench the fist, as this procedure may lead to spurious haemolysis and/or hyperkalaemia (*53*).

### Tube type and the order of draw

Collecting blood in the appropriate tubes and with the appropriate order of draw is crucial to avoid additive carryover. Potassium-EDTA contamination of heparin samples may result in spurious hyperkalaemia and low concentrations of calcium due to EDTA sequestration (*4*). Contamination is reported in up to 0.02% of samples received (*43*). Although following the order of draw is recommended (*40*), it seems that the risk of contamination has become negligible, especially if closed loop systems are used and recommendations of blood sampling are strictly followed (*54*, *55*).

The use of inappropriate containers accounts for 0.03% to 3.6% of overall sample, or 2.6% to 8.1% of all errors analysed (*6*, *7*, *43*, *44*). Beside the adoption of educational interventions, this error could be avoided by using automated samples labelling systems (*45*).

### Tube filling and mixing

Tubes need to be filled up to the indicated volume, inverted once immediately after blood collection and at least five to ten times, as indicated by manufacturers, at the end of the phlebotomy procedure (*40*). This practice will prevent rejection of specimen due to clotting. Especially for coagulation assays, correct filling of tubes is essential to ensure an adequate blood/citrate-ratio. The results of the activated partial thromboplastin time (APTT) may already be biased in samples filled to ≤ 90% of the theoretical filling volume (*56*). In the EFLM WG-PRE observational study, tubes were under- or overfilled in 24.2% of cases (*42*). Laboratories report a wide range of rejected samples due to insufficient sample volume or inadequate sample-anticoagulant ratio (*i.e.*, between 0 - 34.9%), accounting for up to 13.1% of all errors (*7*, *34*, *43*, *44*).

The aforementioned observational study revealed that 30.4% sample tubes were not correctly mixed (*42*). Rejection rates due to clotted specimen are reported to involve up to 27.9% of samples (*34*, *43*, *44*).

To ensure the right sample collection it is necessary to standardize this procedure and organize trainings and audits for all involved members of the healthcare staff regularly (*22*, *45*). Since the adherence to available recommendations seems to be low, the laboratory should provide local indications, for example based on guidelines of the EFLM WG-PRE and COLABIOCLI WG-PRE-LATAM, the CLSI or the World Health Organization (WHO), in the national language and establish a system to guarantee that all phlebotomists are trained correctly (*40*, *42*, *52*, *57*). For implementation and maintenance of such a system, the EFLM WG-PRE provides guidelines in several languages, as well as freely accessible tools, available at https://www.eflm.eu/site/page/a/1194. In addition, the laboratory should monitor the quality of blood collection by evaluation of appropriate QIs. These may reflect the number of samples with misidentification, incorrect sample type, incorrect filling volume, clotting or inappropriate time in sample collection, when appropriate (*e.g.* for circadian hormones and proteins) (*18*).

## Sample/patient identification

Identification errors may occur at several steps of the TTP, and are mentioned in the respective chapters (test ordering/test selection, sample collection, sample preparation and test reporting).

## Transport

The analytical stability of analytes is highly dependent on the time passed between blood collection and analysis, as well as on temperature and other ambient conditions (*i.e.* light exposure). Whereas some parameters may be stable for a long time, others may already be altered one hour after blood collection, or even earlier (*58*). To ensure the right sample transportation, local requirements have to be defined and distributed to all clinicians, nursing staff and carriers (*22*, *59*). A survey among European laboratories on preanalytical practices for coagulation tests recently found that only 42% of participating laboratories are actually monitoring temperature during transportation (*60*). Reported proportions of unsuitable samples due to inappropriate time and temperature conditions can be as high as 3.4% and 1.2% of all samples received (Figure 5), respectively, and 1.9% in relation to the errors analysed (Figure 4) (*7*, *44*). Unsuitable samples concerning transportation or storage should be monitored as QIs, and data loggers for time and temperature tracking may collect objective information for sample acceptance or rejection, as well as for recognizing and improving transportation errors (*18*, *61*).

Beside deviations in time and temperature, samples can also get lost or damaged during transportation, and these events account for 0.2% of samples or 3.1% of all errors analysed (*7*, *18*, *44*).

The use of pneumatic tubes systems (PTS) for sample transportation is commonplace in many hospitals (*59*). This type of sample delivery has been shown to induce cellular rupture of fragile blood cells, thereby potentially biasing test results (*62*, *63*). However, because acceleration vector sums, peak g-forces, length and temperature depend on the construction and use of each specific PTS, studies show a high degree of heterogeneity (*62*). Therefore, each laboratory should validate the local PTS by monitoring potentially affected parameters in relation to g-forces recorded by 3-axis accelerometers as Farnsworth *et al.* showed (*63*).

## Sample preparation

After arrival in the laboratory, the sample has to be registered in the laboratory information system (LIS). Subsequently, most samples need to be centrifuged, decapped, aliquoted and sorted, depending on the requested analytes. Sample integrity has to be assessed whenever analytes are potentially biased by preanalytical variables such as underfilling, HIL, clots or air bubbles (*45*, *64*). Centrifugation of serum samples need to be delayed until clot formation is completed, otherwise fibrin strands may clog the pipetting needle, so leading to inaccurate aspiration and even temporary malfunction of the analyzer (*59*).

Error rates and QIs for unsuitable filling volume and clotted specimens have already been mentioned in the chapter “Sample collection”. Transcription errors may occur in facilities not using electronic order-entry systems (*33*). In general, every step of sample preparation which can be automated by pre-analytical workstations is effective to mitigate the risk of human errors (*33*, *45*, *64*).

### Haemolysis, icterus and lipemia

Haemolysis, icterus and lipemia may lead to erroneous test results of several analytes due to physical and chemical interferences (*65*-*67*). The assessment of so-called HIL-indices by spectrophotometric measurements should always be preferred over visual estimations (*65*, *66*, *68*). Parameter-specific HIL-cut-off values for sample rejection are mostly provided by manufacturers, but should then be verified by the laboratory before being implemented (*65*, *68*, *69*).

The results of a survey among 1405 European laboratories show that 14% of responders do not regularly monitor HIL and 30% state to assess HIL interference only by visual inspection (Figure 2) (*70*). Haemolysis is reported in up to 2.2% of all samples received in clinical laboratories (Figure 5) (*34*, *43*, *44*). However, when blood collection is performed using intravenous (IV) catheters, haemolysis rates may grow substantially. Wollowitz *et al.* reported overall haemolysis rates for blood collection through butterfly needles and IV catheters of 2.7% and 14.6%, respectively (*71*). In the majority of cases haemolysis occurs *in vitro* and may therefore be prevented at several steps of the testing process from collection (*e.g.* using of appropriate needles or low vacuum tubes, avoiding excessive shaking) to transport (*e.g.* ensuring appropriate transport conditions), and sample preparation (*e.g.* appropriate force and time of centrifugation) (*65*, *67*, *72*). Monitoring of haemolysed samples by measurement of relative QIs is highly recommended (*18*).

Unlike haemolysis, lipemia and icterus may be considered *in vivo* interferences. Lipemia account for approximately 0.1 - 2.5% of all samples rejected (*44*, *66*). To prevent lipemia, blood sampling should not be performed after eating a meal or intravenous administration of lipid emulsions. When the presence of lipemia cannot be eliminated, additional centrifugation steps, sample dilution or specific clearing reagents might be helpful (*66*).

### Centrifugation

Since recommendations on centrifugation conditions from manufacturers of blood collection systems as well as other sources differ in time and speed, ranging from ≤ 1300xg to 4000xg, and from 3 to 15 minutes), this preanalytical step may display large heterogeneity (*58*, *73*-*75*). A survey carried out by the External Quality Assurance Providers in Laboratory Medicine (EQALM) recently confirmed that this practice is considerably variable across many European laboratories (*60*). Since blood tubes manufacturers cannot validate all available parameters on all analytical platforms, recommendations are mostly set to a longer centrifugation time at a lower speed to assure sample quality. However, several studies showed that shorter centrifugation time at a higher speed may not significantly alter specific tests results, while being effective to lower the turnaround time (*76*, *77*).

## Analysis

### Stability of parameters

To assure that analyses are carried out within the predefined time of stability of the various laboratory parameters, all necessary timestamps, such as specimen collection time, time of centrifugation and analysis must be available in the LIS (*59*). As already mentioned above (see sections Time of blood collection, Transport), automated systems for linking barcodes of patients and tubes or data loggers could aid in documenting timestamps (*33*, *45*, *61*).

### Quality control in the analytical phase

The internal quality control (IQC), as well as EQA schemes, are cornerstones of quality assessment in the analytical phase (*78*, *79*). Laboratories must ensure that results cannot be released when internal quality control is out of range, as this mistake is reported by Carraro *et al.* (Figure 4) (*7*). Quality indicators encompass unacceptable quality control (QC) results as well as the number of tests uncovered by QC (*18*).

Despite a high degree of standardization and implementation of quality management systems, errors in the analytical phase can still be operator-dependent, a consequence of deviations from recommendation/guidelines or attributable to instrumental malfunctioning (Figure 4) (*6*, *80*).

### Analytical interferences

Analytical interferences are still a huge challenge. As previously mentioned, well-known and “prior-to-analysis” measureable interferences (*e.g.*, HIL), which are also referred to as type 1 interferences, should be checked by automated HIL assessment (*65*, *66*, *68*, *81*, *82*).

The so-called type 2 interferences can be due to heterophilic antibodies, anti-animal antibodies, anti-reagent antibodies, rheumatoid factor, biotin, macrocomplexes or paraproteins (*81*-*83*). Immunoassay results can be altered by such interferences in up to 4% (Figure 1) (*84*). Even if Emerson *et al.* cite in their review that the incidence of interference is estimated to be < 2%, the risk of errors is unquestionably higher considering the large number of immunoassays routinely performed in clinical laboratories (*81*). Because these interferences are reproducible, occur unexpectedly, and cannot be detected by conventional quality control procedures, the possibility of false-positive or false-negative results must always be taken into account, especially if plausibility or delta-checks are suspicious. Nevertheless, interferences may even be clinically plausible, thus making their identification really challenging (*84*). Upon suspicion, algorithms may help to detect analytical interferences by several measures (Appendix 1) (*82*, *83*). Information about analytical interferences should be included in the patient’s medical report since interfering antibodies may persist for a long time.

Notably, some conditions of the specimen itself may also lead to invalid test results. Extreme leucocytosis can lead to pseudohyperkalaemia or pseudohypoglycemia (*5*, *85*). Hyperlipidemia or hyperproteinaemia result in spurious pseudohyponatremia when indirect ion-selective electrodes are used for measurement (*85*, *86*). In cases of implausible results, re-testing needs to be conducted.

### Result transfer

After analysis, results have to be entered into the LIS. Plebani *et al.* reported that 2.6% of mistakes were related to transcription errors (*6*). Since manual procedures seem to be especially vulnerable to clerical errors, automation of result transfer from analyzer to LIS should be preferred, and manual transcription errors monitored (*18*, *33*, *80*).

## Test reporting

### Evaluation/validation of test results

The process of deciding whether or not a result can be released comprises the comparison with reference intervals, critical values or clinical decision limits, as well as the assessment of delta-checks, taking clinical diagnosis and therapeutic procedures into account in order to enhance the possibility of detecting preanalytical or analytical errors that have been undetected so far (*33*, *87*). In general, doubtful results should be replaced by a comment providing appropriate information and recommendation for further sample management (*i.e.* recollection) (*87*). However, considering the potential clinical importance of knowingly biased results (*i.e.* due to haemolysis), Lippi *et al.* proposed an alternative approach. These values may be released accompanied by a comment, when the deviation of the test results is unlikely to exceed clinical significance, which may be assessed with calculation of the reference change value (RCV) (*68*).

Up to 85% of reported identification errors may be noticed before results are made available to clinicians or patients, once again highlighting the importance of correct patient result validation (*22*, *88*). Automated validation systems, which have proven to be efficient, may partly replace manual validation, a time-consuming and almost subjective task (*89*, *90*). However, these systems must be validated to prevent the release of erroneous test results (*87*).

### Reference intervals, decision limits and reference change value

On the laboratory report, results must be provided with the correct measuring unit, preferentially SI units (*22*, *87*). In addition, the appropriate reference intervals (RI) or decision limits (DL), taking into account age and gender of the population must be provided for appropriate data interpretation (*87*, *91*). However, it must be kept in mind, that RIs only cover the central 95% of the studied population.

Since RIs determination for the local population and the specific analytical methods carried out in 120 healthy subjects for each age-range, race and gender, is time-consuming and financially unsustainable by many laboratories, verification of already published reference intervals seems to be a feasible solution (*92*). For further information we refer to CLSI document C28-A3c or the recommendation of the Working Group Accreditation and ISO/CEN standards (WG-A/ISO) of the EFLM (*92*, *93*).

Alternatively, DLs, which are established by consensus for lipids and glycated haemoglobin (HbA1c), may be provided for interpretation. Laboratories may verify the correct use of RIs and DLs by the relative quality specifications, as proposed by Ceriotti *et al*. (*91*). However, the concept of such RI does not take preceding values into account. The RCV has hence been proposed for better reflecting the (clinically) significant change of serial results (*94*).

### Critical values

According to ISO 15189 requirements and other recommendations, critical values must be clearly defined, along with a detailed process on how stakeholders will be informed in a timely manner (*13*, *87*). Although there are differences in the way clinical laboratories report critical values, a survey among Croatian medical biochemistry laboratories showed that 99.1% of responders follow these requirements (*95*). Thresholds beyond which test results are considered critical need to be defined based on well-designed outcome studies and in collaboration with clinicians. However, such thresholds are often outdated or based on expert opinion, as reliable studies are often missing (*96*). The time from result validation to result communication should be documented and benchmarked as QI for purposes of future improvement (*18*, *87*).

### Turnaround time

To avoid delays in diagnosis and treatment, patient samples should be processed as quickly as possible (*22*). However, different approaches to measurement and definition of turnaround time (TAT) makes data comparison often challenging (*97*). The total TAT or “therapeutic TAT”, describes the time interval between test ordering to the time when a treatment decision is made. As timestamps necessary to calculate these intervals are often missing (see chapters Sample collection and Transport), laboratories often refer to the intra-laboratory TAT, intended as the time from sample reception in the laboratory to release of test results. These time intervals should be collected on a regular basis for surrogate parameters and benchmarked with other laboratories (*18*). If the results do not meet the target values, total laboratory automation may help improving TAT (*64*). In addition, intra-laboratory sample processing should be continuously monitored with color-coded alarms when individual samples are processed too slow.

Although the measurement of intra-laboratory TAT is easier, laboratories should aim to collect data on therapeutic TAT, since up to 96% of delays are non-analytical (*97*). Again, collaboration with clinicians is necessary to understand their expectations and to assess where improvements outside the laboratory are most feasible.

### Report correction

Despite thoughtful validation, errors may be detected after the report has already been made available to clinicians or patients. In a retrospective analysis, only 0.01% of the reported results had to be corrected (Figure 1) (*80*). Nevertheless, each revised result involves the risk of being overlooked by the physician (Figure 1 and 4) (*6*, *80*). Therefore, the responsible persons must be informed whenever laboratory reports were changed (*80*, *87*). The above-mentioned study revealed that this procedure has only been documented in 58% of cases. The number as well as the reasons of revised reports should be assessed to identify and improve error-prone steps throughout the TTP (*18*, *80*, *87*).

## Interpretation and action

### Acknowledgement of test results and patient communication

Physicians may receive up to 1000 laboratory test results each week (*98*). Ideally, a visualization technique that meets the local requirements is selected in collaboration with clinicians, for ensuring appropriate assessment of crucial results. A survey focusing on potentially actionable results, which were not available at the patient’s discharge, revealed that the rate of unawareness was as high as 61.6% (Figure 2). Thereof 37.1% of cases would have required diagnostic or therapeutic alterations, whilst urgent action would have been necessary in 12.6% of cases (*99*).

Patients should also be informed on test results, diagnoses as well as changes in therapy. An evaluation in an outpatient clinic, carried out by Schiff *et al.*, revealed that 2% of potassium prescriptions were related to patients whose current or previous potassium result was ≥ 5.3 mmol/L, and that no evidence of contacting the patient could be found (*100*). In another study Schiff *et al.* found that at least 2% of patients with thyroid-stimulating hormone concentration of ≥ 20 mIU/mL were not informed about their pathologic result or a potential diagnosis of hypothyroidism (*101*). The failure rates to inform patients about abnormal results or to document this action are found to be as high as 26.2% (Figure 1) (*102*). Reporting results in addition directly to the patient may be one possibility to reduce missed diagnoses. However, the advantages and disadvantages have to be considered before implementation (*103*).

### Interpretation

Providing laboratory test results in the fastest time and with the highest possible quality may both be useless when data are incorrectly interpreted. Interpretation of laboratory test results has to be performed considering clinical history, symptoms, physical examination and results of other diagnostic disciplines, so deciding whether or not the test result is valid and eligible for patient care (*104*).

According to a survey carried out by Hickner *et al.*, nearly 8% of primary care physicians may have uncertainties in interpreting laboratory test results, but even laboratory staff may be challenged in specific situations (*11*, *105*). Gandhi *et al.*, along with Kachalia *et al.*, evaluated closed malpractice claims and reported that incorrect interpretations may account for up to 37% of missed or delayed diagnoses (Figure 3) (*25*, *26*). At least 50% of reported erroneous results due to analytical interference, which were not recognized in the process of validation, led to misdiagnosis and inappropriate management by the clinician (*83*).

Furthermore, different analytical methods or instruments often provide non-comparable results of the same analyte (*e.g.* hormone tests) (*106*). In addition, clinical conditions may bias test results, *e.g.*, HbA1c test results may be falsely low when underlying diseases are associated with a reduced erythrocyte lifespan (*85*). Other biasing conditions include unchangeable interferences of the specimen itself, such as leucocytosis or hyperproteinaemia (see chapter Analysis), or deviations from recommendations on blood collection (see chapter Sample collection). All these circumstances need to be acknowledged and taken into account when interpreting test results.

Since laboratory specialists are trained in the task of test selection and interpretation, they should aid clinicians in diagnosing patients correctly (*107*). Especially when results are not consistent with the clinical picture, physicians should be encouraged to get in touch with the laboratory. However, Hickner *et al.* found that although laboratory consultations are rated as helpful by 35% of surveyed primary care physicians, only 6% approach laboratory professionals in case of uncertainty during test interpretation (*11*).

Reflective testing as well as narrative interpretation of results may aid to reduce medical error (*31*). The latter might even have an educational impact on test selection and ordering behaviour or a positive effect on the health care budget (*28*, *108*). As a premise, clinical information and indications must be provided along with the test request. In addition, diagnostic management teams have proven the same efficiency (*32*, *82*). The number of reports with interpretative comments can be assessed as QI (*18*).

## Conclusion

Since laboratory results are essential in most medical decisions, high quality laboratory testing with an appropriate TAT is crucial. Although several guidelines and recommendations (summarized in Appendix 1) are available for every step in the TTP, an observational study has shown low compliance rates thereof (*42*). Moreover, published data on error rates are still high for the extra-laboratory phases as we could demonstrate in this review.

In our opinion, the core duty of medical laboratories is not only to provide high quality analytics but also to aid in finding the right diagnosis of patients. Therefore, laboratory specialists should make an effort to surveil the whole TTP using the QIs concept (*18*) and refocus on improving error-prone extra-laboratory processes, especially test selection and interpretation, in collaboration with physicians of other medical departments (*107*).

## Appendix 1 Overview of the total testing process concerning potential errors, quality indicators and possible solutions for improvement

**Table:**

**TTP phase**	**TTP step**	**Sources of error**	**QI/measurement (18)**	**Possible solutions for improvement**
**Preanalytical phase**	Test ordering/ test selection	Overutilization	Inappropriate test requests	Education through feedback (10) Interpretive comments (28) Automated flags (24) Gate-keeping strategies (29) Diagnostic pathways (“algorithms”) (30) Reflex criteria (8) Reflective testing (31) Diagnostic management teams (32)
		Underutilization		
		Order is misinterpreted or unintelligible	Misidentification error Test transcription errors Unintelligible requests	Computerized physician order entry system (33,35)
		Erroneous information on the request		
	Sample collection/ venous blood sampling	Patient identification	Misidentification error	Implementation of recommendations/ guidelines (40,52,57) Use at least two identifiers (33,41) Ask open questions, mind the patient’s identification bracelet (41) Barcoding system (33,35) Automated systems for labelling of tubes/pre-labelled tubes (33,45) Education (22,45)
		Patient preparation	Inappropriate time in sample collection	
		Inappropriate container	Incorrect sample type	
		Tube filling	Incorrect fill level	
		Tube mixing	Clotted samples	
	Sample identification	Misidentification	Misidentification errors	Pre-analytical workstations (33,45,64) Delta-check alerts at the stage of validation (33,87)
	Transport	Time and Temperature	Unsuitable samples for transportation and storage problems	Define and distribute information about local requirements (59) Use data logger for time and temperature (61)
		Damaged or lost samples		
		Pneumatic tube system	/	Validate pneumatic tube system (63)
	Sample preparation	Aliquotting	/	Automatization (33,45)
		Hemolysis	Haemolysed sample	Assessment by spectrophotometric measurement (65,68) Prevent hemolysis (65,67,72) German/Austrian Preanalytical Benchmark Database (19)
		Icterus / Lipemia	/	Assessment by spectrophotometric measurement (65,66)
**TTP phase**	**TTP step**	**Sources of error**	**QI/measurement (18)**	**Possible solutions for improvement**
**Analytical phase**	Analysis	QC	Test uncovered or unacceptable performances	QC management (78,79)
		Type 2 interferences of immunoassays	/	Algorithms including several measures like re-testing using different methods, serial dilutions to reveal nonlinearity, polyethylene glycol precipitation procedures or pretreating specimens with blocking reagents (82,83)
		Transcription of results	Data transcription errors	Automation of result transfer (33)
**Postanalytical phase**	Test reporting	Validation	Misidentification error	Take into account: reference intervals, critical values or clinical decision limits, delta-checks, clinical diagnosis and therapeutic procedures (33,87) Automated validation systems (89,90)
		Reference intervals or decision limits	/	Determination / verification (92,93) Correct use (91)
		Critical results	Notification of critical results	/
		Turnaround time	Inappropriate turnaround times	Total laboratory automation and color-coded alarms (64)
		Revised Results	Incorrect laboratory reports (18, 87) Reasons of rectified results (87)	Information about amended reports (80,87)
	Interpretation and action	Unawareness of results	/	Reporting results in addition directly to the patient (103)
		Patient not informed		
		Interpretation (incorrect, uncertainty)	Interpretative comments	Interpretative comments (28,31,108) Reflective testing (31) Laboratory diagnostic pathways (30) Diagnostic management teams (32,82)
TTP – total testing process. QI – quality indicators. QC – quality control. The numbers in brackets indicate references.				
